# Commentary: Potential Mechanism Prediction of Herbal Medicine for Pulmonary Fibrosis Associated With SARS-CoV-2 Infection Based on Network Analysis and Molecular Docking

**DOI:** 10.3389/fphar.2021.781941

**Published:** 2021-11-02

**Authors:** Congchao Jia, Xinya Peng, Hao Chi, Gang Tian

**Affiliations:** ^1^ Clinical Medical College, Southwest Medical University, Luzhou, China; ^2^ Department of Laboratory Medicine, The Affiliated Hospital of Southwest Medical University, Luzhou, China

**Keywords:** COVID-19, network pharmacology, pulmonary fibrosis, molecular docking, Chinese herbal medicine

The coronavirus disease 2019 (COVID-19) emerged as a global pandemic, and by September 23, 2021, it brought the cumulative numbers of confirmed cases and deaths globally to more than 230 million and over 4.7 million, respectively. Though the COVID-19 vaccine is available, effectively controlling the rapid spread of COVID-19 and treating the rising number of diagnosed patients has become an urgent worldwide problem to be solved. Thus, it is urgent to develop safe and effective drugs for treating COVID-19. We read the recent article by (Jin et al., 2021) hopefully, which mainly discovered the multi-component, multi-target, multi-pathway mechanism of Convalescent Chinese Prescription (CCP) treatment for COVID-19 pulmonary fibrosis associated with SARS-CoV-2. These findings offer a new perspective on the treatment of COVID-19, but several key issues need to be addressed.

First, there are several questions about the origin of the components and targets of CCP. TCMSP, BATMAN-TCM were all updated earlier than December 2016 and it is questionable whether overly lagging knowledge can facilitate mechanistic investigations of CCP against COVID-19 (Jiang et al., 2020). In addition, Jin et al. used the TCMSP and Swiss TargetPrediction databases for target fishing. It was reported that Swiss TargetPrediction could only achieve at least one correct human target in the top 15 predictions for >70% of external compounds (Daina, Michielin, & Zoete, 2019). Therefore, the component-target interactions predicted by these databases should be validated by molecular dynamics simulation and experiments. Obviously, it is highly implausible because of no experiment in this study.

Secondly, targets of pulmonary fibrosis associated with SARS-CoV-2 are inaccurate. Pulmonary fibrosis is a chronic, progressive process in which healthy tissues are replaced by altered extracellular matrix and alveolar structures are destroyed, resulting in decreased lung compliance, interrupted gas exchange, and respiratory failure and death ultimately (Richeldi, Collard, & Jones, 2017). We admit that pulmonary fibrosis associated with SARS-CoV-2 has some commonalities with pulmonary fibrosis associated with other causes or idiopathic pulmonary fibrosis, but pulmonary fibrosis associated with SARS-CoV-2 also has its characteristics. Unfortunately, Jin et al. did not notice this, and they roughly collected the targets related to pulmonary fibrosis based on the OMIM database, drug bank database, and the DisGeNET database.

Then, in the GO biological process and KEGG pathway enrichment section, the enrichment strategy is unclear and the results are unreliable. Four of the top five of the KEGG pathway (Pathways in cancer, Pancreatic cancer, Bladder cancer, Colorectal cancer) are associated with cancer, which is not relevant to what we are studying.

At the molecular docking part, there are also some problems. Molecular docking is a process through which small molecules are docked into the macromolecular structures for scoring their complementary values at the binding sites (Saikia & Bordoloi, 2019). The reliability of molecular docking depends on the accuracy of the adopted scoring function, and the scoring function can be used to determine the binding mode and site of a ligand, predict binding affinity and identify the potential drug leads for a given protein target (Li, Fu, & Zhang, 2019). However, there is no universal scoring algorithm for each molecular docking calculation (Cheng, Li, Li, Liu, & Wang, 2009; Li et al., 2019; Saikia & Bordoloi, 2019). Moreover, the docking score is only a relative reference value, and there is no literature supporting that the docking score is greater than three was considered a stable compound binding to the protein.

Finally, the active components identified by the author, such as quercetin, kaempferol, luteolin, are widely distributed in a variety of plants, and a large number of network pharmacology studies have identified quercetin, kaempferol, and luteolin, among others, as candidate molecules for COVID-19 (Huang, Bai, He, Xie, & Zhou, 2020; Xia et al., 2020; Gu et al., 2021; Niu et al., 2021; Ye et al., 2021). It is highly unreliable. The same molecules being repeatedly found may not indicate an actual result, but instead, they may be due to non-specific interactions by particular molecules. The small molecules above are pan-assay interference compounds, and further experiments are needed to determine whether they have anti-pulmonary fibrosis effects.

COVID-19 remains a global public health problem that places a heavy burden on humanity. The development of anti-COVID-19 drugs is urgent, but more attention should be paid to the issues discussed above.
